# Effect of Elasticity on Heat and Mass Transfer of Highly Viscous Non-Newtonian Fluids Flow in Circular Pipes

**DOI:** 10.3390/polym17101393

**Published:** 2025-05-19

**Authors:** Xuesong Wang, Xiaoyi Qiu, Xincheng Zhang, Ling Zhao, Zhenhao Xi

**Affiliations:** 1 State Key Laboratory of Chemical Engineering and Low-Carbon Technology, School of Chemical Engineering, East China University of Science and Technology, Shanghai 200237, China; xuesongwang99999@163.com (X.W.); ss-xiaoyi-ss@163.com (X.Q.); y11210007@mail.ecust.edu.cn (X.Z.); zhaoling@ecust.edu.cn (L.Z.); 2 Shanghai Key Laboratory of Multiphase Materials Chemical Engineering, East China University of Science and Technology, Shanghai 200237, China

**Keywords:** viscoelasticity, constitutive rheological equation, laminar flow, heat and mass transfer CFD simulation

## Abstract

The viscoelasticity of fluids have a significant impact on the process of heat and mass transfer, which directly affects the efficiency and quality, especially for highly viscous functional polymer materials. In this work, the effect of elasticity on hydrodynamic behavior of pipe flow for highly viscous non-Newtonian fluids was studied using viscoelastic polyolefin elastomer (POE). Two constitutive rheological equations, the Cross model and Wagner model, were applied to describe the rheological behavior of typical POE melts, which have been embedded with computational fluid dynamics (CFD) simulation of the laminar pipe flow through the user-defined function (UDF) method. The influence of both viscosity and elasticity of a polymer melt on the flow mixing and heat transfer behavior has been systematically studied. The results show that the elastic effect makes a relative larger velocity gradient in the radial direction and the thicker boundary layer near pipe wall under the same feed flow rate. That leads to the higher pressure drop and more complex residence time distribution with the longer residence time near the wall but shorter residence time in the center. Under the same conditionals, the pipeline pressure drop of the viscoelastic fluid is several times or even tens of times greater than that of the viscous fluid. When the inlet velocity increases from 0.0001 m/s to 0.01 m/s, the difference in boundary layer thickness between the viscoelastic fluid and viscous fluid increases from 3% to 12%. Similarly, the radial temperature gradient of viscoelastic fluids is also relatively high. When the inlet velocity is 0.0001 m/s, the radial temperature difference of the viscoelastic fluid is about 40% higher than that of viscous fluid. Besides that, the influence of elasticity deteriorates the mixing effect of the SK type static mixer on the laminar pipe flow of highly viscous non-Newtonian fluids. Correspondingly, the accuracy of the simulation results was verified by comparing the pressure drop data from pipeline hydrodynamic experiments.

## 1. Introduction

Highly viscous fluids are more and more encountered in the modern industrial field, such as polymer melts and solutions, petroleum, slurry, and paints [1,2,3]. The flow process of highly viscous fluids inside pipes typically occurs in a laminar state characterized by a low Reynolds number and low shear rates, resulting in a relatively thicker flow boundary layer and even some dead zones [4,5]. The low turbulence in the boundary layer will cause great challenge in mixing the fluid near the wall with the fluid in the center of the pipe, which extremely affect the heat and mass transfer efficiency and residence time distribution of the laminar fluid flow. Obviously, that may even lead to a wider molecular weight distribution and poorer quality of highly viscous functional polymer materials.

However, polymer solutions/melts also have elasticity, except viscosity [6,7,8,9,10]. Since rheological characteristics are related to the flow history, viscoelastic fluids have many different flow characteristics from a viscous fluid, such as extrusion swell [11], tubeless siphon [12] and climbing rod effects [13], etc. The effect of elasticity usually cannot be ignored on the flow behavior, pressure drop, and heat and mass transfer [14]. In order to study the flow of viscoelastic fluid, it is very important to select a suitable constitutive equation to describe its rheological behavior [15]. The constitutive equations describing viscoelasticity are mostly based on differential or integral form, and the integral form can better predict the time dependence. The constitutive equations of a viscoelastic fluid are divided into linear viscoelastic constitutive equations and nonlinear viscoelastic constitutive equations. The linear viscoelastic constitutive equations are only applicable to the flow field with small deformation, and the flow field with large deformation in the static mixer is more consistent with the nonlinear viscoelastic constitutive equations [16]. Common viscoelastic constitutive equations include Upper combined Maxwell (UCM) [17], Phan Thien Tanner (PTT) [18], Giesekus [18], K-BKZ [19], and White Metzner [20]. Among them, the Kearsley–Bernstein Kearsley Zapas (K-BKZ) equation is the integral one that has been most widely used [21]. On the basis of the K-BKZ constitutive equation, Wagner et al. studied the variation of stress with time under various flow conditions and proposed a new model, the so-called “Wagner model,” for describing the nonlinear viscoelastic behavior of polymers [22].

There have been some studies on the influence of elasticity on the flow state. Feng [23] investigated the disturbance flow generated by the oscillatory motion of a solid particle in linear viscoelastic (LVE) fluids. They found that there is a sequence of eddies produced in LVE fluids instead of a single one as in the Newtonian fluids. The eddies develop in the interior of the LVE fluids and barely travel, while in the Newtonian fluid, the eddy is generated on the particle surface and propagates into the fluid. Nazemi et al. [24] studied the effects of the viscoelastic properties of non-Newtonian fluids on the flat plate boundary layer numerically. They found that the boundary layer thickness, surface shear stress, and drag coefficient of viscoelastic fluids are less than Newtonian fluids. A model describing the thinning of a free liquid film was developed for viscoelastic liquids by Gambaryan-Roisman [25]. He found out that the film-thinning rate increases with increasing relaxation time. Guillermo et al. [26] studied the drag reduction by polymer addition. The results showed that the addition of polymers regardless of their molecular weight in the laminar flow regime in the pipeline causes no change in power dissipation. Faroughi et al. [27] studied the effects of elasticity on the efficient transport of solid particles using polymeric fluids. They found that there is a reduction in the particle drag coefficient at low levels of elasticity (Wi < 1) and a considerable enhancement at high levels of elasticity (Wi > 1).

In fact, the characteristics and development behavior of the laminar boundary layer are crucial for the heat and mass transfer process, especially for highly viscoelastic fluids. Generally, reducing the thickness of the laminar boundary layer can significantly improve the heat and mass transfer behavior. Increasing the flow rate of a highly viscoelastic fluid can reduce the thickness of laminar boundary layer and increase the corresponding surface heat transfer coefficient, but it will also cause greater resistance loss and energy consumption of the system [28]. Disturbing the fluid near the wall can also reduce the thickness of the laminar boundary layer. There are active and passive methods to disturb the fluid in the pipeline and enhance fluid mixing and heat transfer [29]. The active methods require external energy input, but it will lead to high energy consumption (high viscosity fluids) or will degrade shear-sensitive fluids (such as polylactic acid). The passive method does not utilize moving parts, and the most common passive method is the static mixer. A static mixer is a static element inserted into the pipe to change the direction of fluid flow, which forces fluid mixing and heat transfer to be more uniform. It has many advantages, such as narrow residence time distribution, a large interface area, low maintenance costs, and easy installation [30]. The static mixers have lower investment and operation costs and are more competitive compared with mechanical mixers in industrial applications.

There have been some studies on the flow of highly viscoelastic fluids in static mixers. Migliozzi et al. [31] studied the effects of pure elasticity and the combination of elasticity and shear thinning on mixing dynamics, respectively, through experiments in Kenics and SMB-R static mixers. The results show that the solid-like behavior of viscoelastic fluids will lead to a decrease in mixing quality. Ramsay [32] studied the mixing effect of a Newtonian fluid and viscoelastic fluid in Kenics KM static mixer through experiments. At a given speed, there are significant differences between the mixing mode of Newtonian fluids and elastic fluids. Arratia et al. [33] found that when the viscosity of elastic fluid is similar to that of a Newtonian fluid, the elasticity will slightly inhibit the stretching and mixing of fluids. Caserta et al. [34] studied the mixing of a viscoelastic fluid and Newtonian fluid in the Kenics static mixer and found that viscoelasticity had little effect on the mixing effect. However, there are few reports on the effect of elasticity on the laminar boundary layer in pipes. Meanwhile, apart from the experimental methods, there is still a lack of accurate and efficient simulation methods to explore the influence of viscoelasticity on heat and mass transfer in pipeline flow.

In this work, a typical viscoelastic polyolefin elastomer (POE) melt was used as a highly viscous medium to explore the influence of viscoelasticity on heat and mass transfer in pipeline flow. Two non-Newtonian fluid constitutive equations, the Cross model and Wagner model, were applied to describe the viscoelastic behavior of the POE melt. Two constitutive rheological equations have been embedded with a computational fluid dynamic (CFD) simulation of the laminar pipe flow through the UDF method. The impact of both viscosity and elasticity on the pressure drop, velocity field, heat, and mass transfer of laminar flow inside the pipe with a highly viscous non-Newtonian fluid have been simulated and explored in detail. The velocity boundary layer and the thermal boundary layer especially have been quantificationally compared. In addition, the difference in the mixing effect between a viscoelastic fluid and viscous fluid was studied in the pipeline with an SK static mixer.

## 2. Materials and Methods

### 2.1. Typical Polymer and Its Rheological Constitutive Equation

A typical viscoelastic polymer is commercial grade of polyolefin elastomer POE8150 produced by Dow and purchased from Beijing Jintong Chemical Company (Beijing, China). POE8150 is a copolymer of ethylene and octene; its density is 0.868 g/cm^3^, and its melting temperature is 55 °C. Before rheological testing, the POE8150 melt particles were made into polymer sheet, and the dimensions were 20 mm in diameter and 2 mm in thickness using Thermo Scientific HAAKE MiniJet Pro (Thermo Fisher Scientific, Waltham, MA, USA). Then, rheological data of POE8150 melt were tested through HAAKE MARS60 rotational rheometer (Thermo Fisher Scientific, Waltham, MA, USA) with parallel plate geometry of Thermo Fisher Scientific (Waltham, MA, USA).

The Cross model of generalized Newtonian fluid constitutive equation without considering elasticity and the Wagner model of viscoelastic constitutive equation were used to fit the rheological data of POE8150.

Cross model is used to describe the viscosity change of the whole shear rate range, and the model contains four parameters. The equation is as follows:

(1) $$\frac{\eta -\eta_{\infty }}{\eta_{0}-\eta_{\infty }}=\frac{1}{1+{\left(\lambda \dot{\gamma }\right)}^{m}}$$

where *η*_0_ is the viscosity of the first Newtonian region; *η*_∞_ is the viscosity of the second Newtonian region; *λ* is the characteristic time, which is directly related to the material properties; and *m* is the fluid behavior index, which is related to the material properties.

Wagner model based on K-BKZ constitutive equation is used to describe the viscoelastic behavior of fluids. The constitutive equation of Wagner is as follows:

(2) $$\tau \left(t\right)=\int_{-\infty }^{t}m\left(t-t^{\prime }\right)h\left(\gamma \right)\gamma \left(t,t^{\prime }\right)dt^{\prime }$$

where *m*(*t* − *t′*) is the memory function, which is time derivative of relaxation modulus; *h*(*γ*) is referred to as the “damping function”, which can be used for describing the nonlinear rheological response; and *γ*(*t*, *t′*) is the deformation history from time *t′* to *t*.

The memory function can be acquired from the curve of stress relaxation modulus in the linear region; it can be represented as

(3) $$m\left(t-t^{\prime }\right)=\sum_{i=1}^{N}\frac{g_{i}}{\lambda_{i}}e^{-\left(t-t^{\prime }\right)/\lambda_{i}}$$

where *g*_i_ is the relaxation modulus, *λ*_i_ is relaxation time, and *N* is the number of the Maxwell models. They can be determined by SAOS test. The relaxation time and relaxation modulus can be obtained from the dynamic modulus of *G*′(*ω*) and *G*″(*ω*). The *N* sets of the relaxation time *λ*_i_ and the relaxation modulus *g*_i_ can be determined by nonlinear regression using following expression:

(4) $$G^{\prime }\left(\omega \right)=\sum_{i=1}^{N}\frac{g_{i}\omega^{2}\lambda_{i}^{2}}{1+\omega^{2}\lambda_{i}^{2}}$$

(5) $$G^{\prime \prime }\left(\omega \right)=\sum_{i=1}^{N}\frac{g_{i}\omega \lambda_{i}}{1+\omega^{2}\lambda_{i}^{2}}$$

The damping function is used to express deviation from linear to nonlinear viscoelastic behavior of the material. It has value between one and zero. The material exhibits a linear viscoelastic behavior when the damping function is one, and the material’s behavior becomes nonlinear as the damping function approaches to zero. It can be determined by the relationship between the linear and nonlinear relaxation modulus, as written in Equation (6).

(6) $$h\left(\gamma \right)=\frac{G\left(t,\gamma \right)}{G\left(t\right)}$$

where *G*(*t*) is the linear stress relaxation modulus and *G*(*t,γ*) is the nonlinear stress relaxation modulus.

Below the critical strain *γ*_c_, the linear stress relaxation modulus *G*(*t*) does not change with strain. Over the critical strain *γ_c_*, the nonlinear stress relaxation modulus *G*(*t,γ*) decreases with the increase in strain. The damping function *h*(*γ*) is obtained by the ratio of nonlinear stress relaxation modulus to linear relaxation modulus.

As for simple shear, Wagner proposed the following expression for the damping function:

(7) $$h\left(\gamma \right)=e^{-\alpha \gamma }$$

Curve fitting was performed on the experimental data to obtain fitting parameter *α* of the Wagner model.

### 2.2. CFD Simulation Details

**Figure 1:**
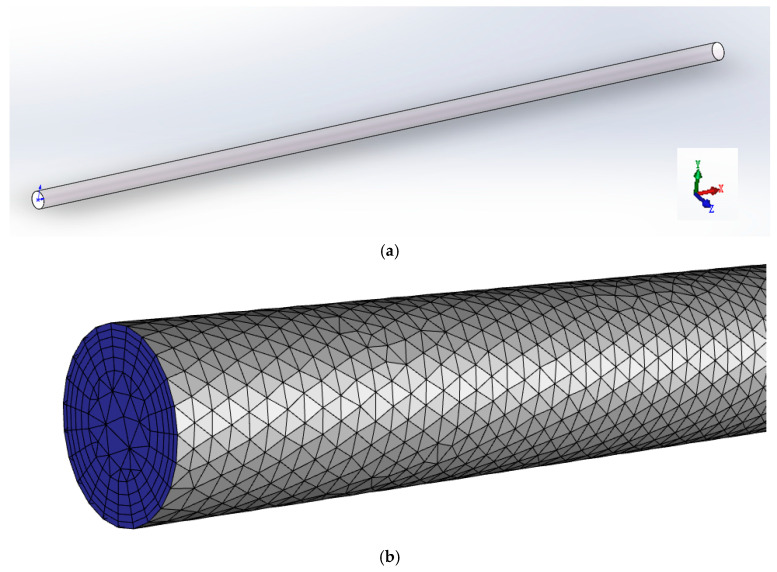
The physical model (**a**) and the grid (**b**) of the empty pipe.

**Figure 2:**
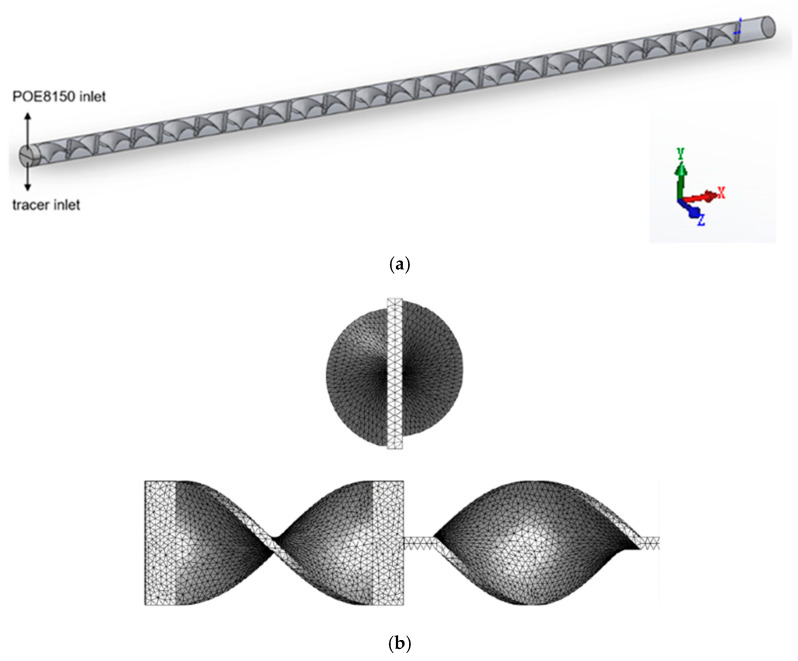
The physical model (**a**) and the grid (**b**) of the SK static mixer.

**Figure 3:**
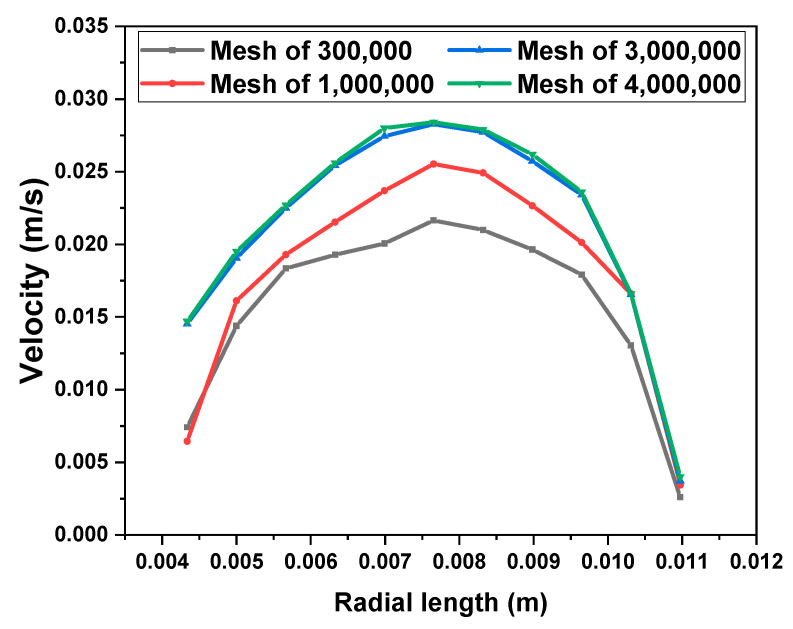
The mesh independence verification of the velocity distribution of the viscoelastic fluid on the radius of the SK static mixer.

**Figure 4:**
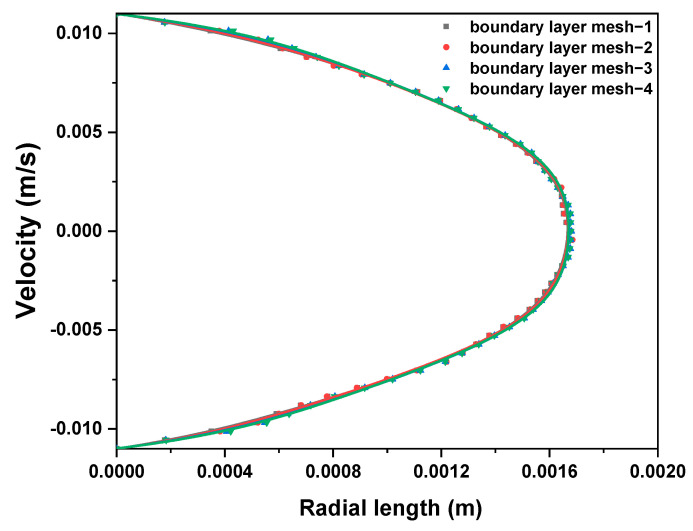
The influence of boundary layer mesh resolution on the velocity distribution of the viscous fluid inside an empty pipe.

The pressure drop, velocity field, and temperature field of viscous fluid and viscoelastic fluid were compared by simulating the flow of the fluids in the heating empty pipe. The mixing efficiency of viscous fluid and viscoelastic fluid was compared by simulating the mixing of the fluids and tracer in the SK static mixer. The length of the pipeline was 1 m, the inner diameter of the pipeline was 22 mm, the diameter of the SK static mixer was 20 mm, the aspect ratio of a single component was 2, and the thickness of the component was 2 mm. The 3D models were established by SOLIDWORKS, and the meshes were generated by ANSYS Meshing 2020 R2; all meshes were tetrahedral. The flow state of the fluids was laminar flow, so boundary layer mesh division was also performed near the wall. The physical model and the grid of the empty pipe were shown in Figure 1. The physical model and the grid of the SK static mixer were shown in Figure 2. Taking the velocity distribution of viscoelastic fluid on the radius of SK static mixer as an example, mesh independence verification was conducted and shown in Figure 3. Taking the radial velocity distribution of viscous fluid in an empty pipe as an example, the influence of boundary layer mesh resolution was verified and shown in Figure 4.

The simulation software was the commercial software FLUENT 2020 R2 of ANSYS. Cross model is included in FLUENT; the relative model parameters can be directly set in the software for simulation. The viscoelastic constitutive equation Wagner model is not included in FLUENT, and it need to be embedded into FLUENT through UDF method. The fluid inlet was defined as the “velocity-inlet”, the fluid outlet was defined as the “pressure-outlet”, and the wall was defined as the “Stationary Wall”, which was no slip. The temperature of the pipe wall was constant, and the heating temperature was 20 °C higher than the inlet temperature of the fluids.

### 2.3. Pipeline Flowing Experiments

**Figure 5:**
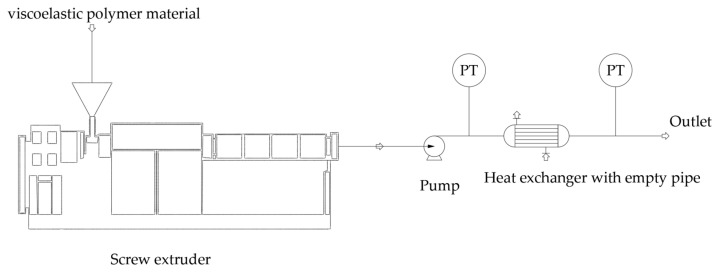
The continuous hot model testing device for polymer fluid (The arrows represent the direction of viscoelastic polymer material flow).

The pipeline flowing experiments were carried out in the continuous hot model testing device with viscoelastic polyolefin elastomer melt as shown in the Figure 5. The POE particles were heated and extruded by a twin-screw extruder (Nanjing Chuangbo Machinery Equipment, Ltd. (Nanjing, China)) and then transported to the heat exchanger by the melt pump. The heat exchanger is a tubular heat exchanger; the length of the empty tube inside the heat exchanger is 1 m; and its diameter can be changed with 15 mm, 20 mm, 25 mm and 33 mm. Pressure sensors and temperature sensors were set at the inlet and outlet of the heat exchanger to measure the temperature and pressure drop of the polymer fluids. The fluid flow rate can be controlled by adjusting the feeding speed of the weight-loss scale equipped on the extruder. The polymer melt temperature in the empty pipe was controlled by adjusting the hot oil temperature of the heat exchanger.

The continuous hot model testing device can effectively collect the pressure drop data of viscoelastic polyolefin elastomer melt passing through the pipeline, which can be compared with the simulation results to verify the accuracy of the simulation results.

## 3. Results and Discussion

### 3.1. Rheological Behavior of POE Melt

**Figure 6:**
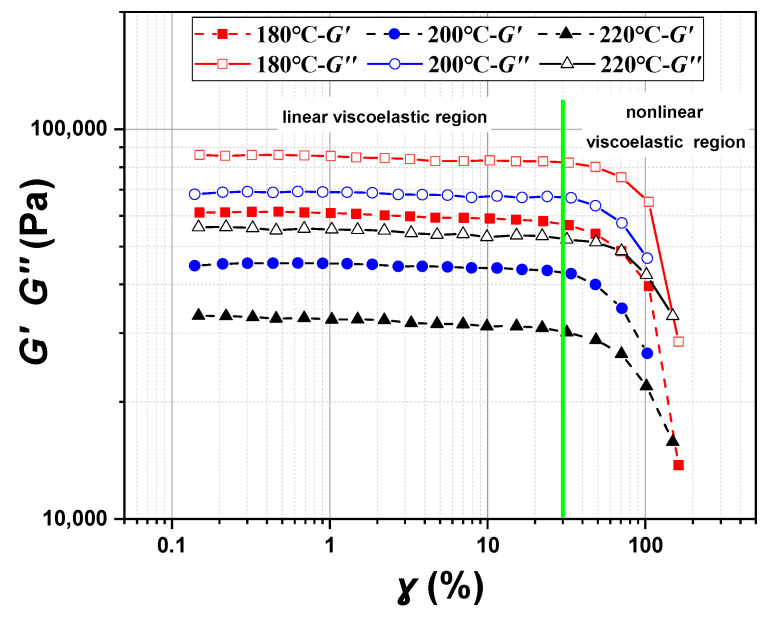
Linear and nonlinear viscoelastic region of POE8150 at 180 °C, 200 °C, and 220 °C.

Because the relationship between the shear strain and the shear rate of different materials is different, it is necessary to clarify the linear viscoelastic region (LVR) of the POE melt. The Oscillation Amplitude Frequency Sweep program is suitable to test the linear viscoelastic region of POE8150. The test temperature is 180 °C~220 °C, and the strain range is 0.1%~150%. The oscillation frequency keeps constant, and the strain changes within a certain range to obtain the variation of modulus with strain. The linear and nonlinear viscoelastic region of POE8150 at different temperatures were shown in Figure 6. The green line is the boundary between linear viscoelastic region and nonlinear viscoelastic region. In the linear viscoelastic region, the modulus basically remains constant. When the shear strain is below 30%, it is the linear viscoelastic region of POE8150.

**Figure 7:**
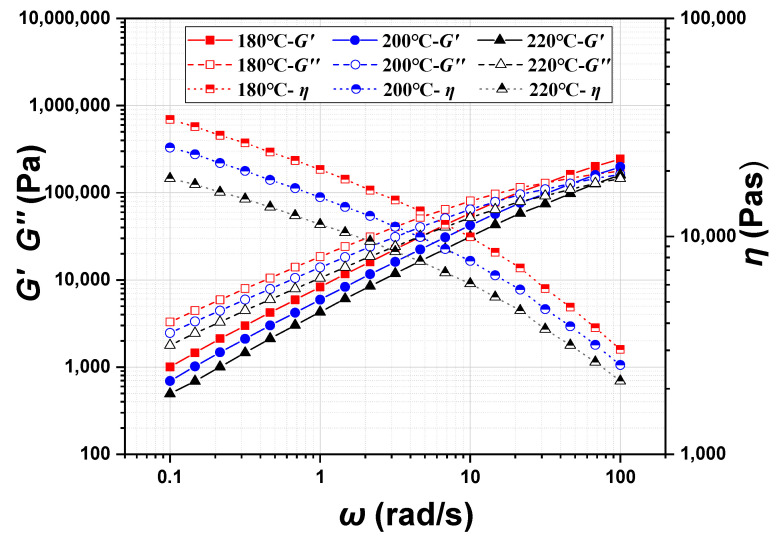
*η*, *G′*, and *G″* of POE8150 at 180 °C, 200 °C, and 220 °C.

The linear viscoelastic properties of the POE melt, such as the viscosity (*η*), the storage modulus (*G′*), and the loss modulus (*G″*), were measured by the Small Amplitude Oscillatory Shear (SAOS) program for wide ranges of frequency and temperature. The SAOS program measures stress or strain as a function of frequency by applying sinusoidal stress or strain to characterize the linear viscoelastic properties of the materials. Since the range of strain is below 30% for the linear viscoelastic region of POE, the strain value in the SAOS testing is set to 0.3%. The test temperature range is 180 °C~220 °C, and the angular frequency range is between 0.1 and 100 rad/s. The test results are shown in Figure 7. The viscosity (*η*) of the POE melt decreases with the increase in oscillation frequency, indicating its shear thinning characteristic. The storage modulus (*G′*) and the loss modulus (*G″*) increase with the increase in oscillation frequency. The lower storage modulus (*G′*) compared to the loss modulus (*G″*) indicates that the POE melt’s Tm is below 180 °C. The viscosity (*η*), the storage modulus (*G′*) and the loss modulus (*G″*) all decrease with the increase in temperature.

The behavior of the polymer materials is linear when the strain is sufficiently small and slow. However, the nonlinear behavior appears with the increase in strain [35]. The parameters of nonlinear viscoelasticity can be measured by the step strain test. The step strain test is used to study the variation in the stress relaxation modulus of materials under different strains over time. In the linear viscoelastic region, the relaxation modulus is only related to time and does not change with the strain, while the relaxation modulus in the nonlinear viscoelastic region is related to time and strain. The Rotation Time Curve program was chosen to carry out the step strain test. By imposing a step-wise shear strain ranging from *γ* = 1% to *γ* = 550% at the temperature of 180 °C, 200 °C, and 220 °C on the POE samples, the stress relaxation modulus was obtained.

**Figure 8:**
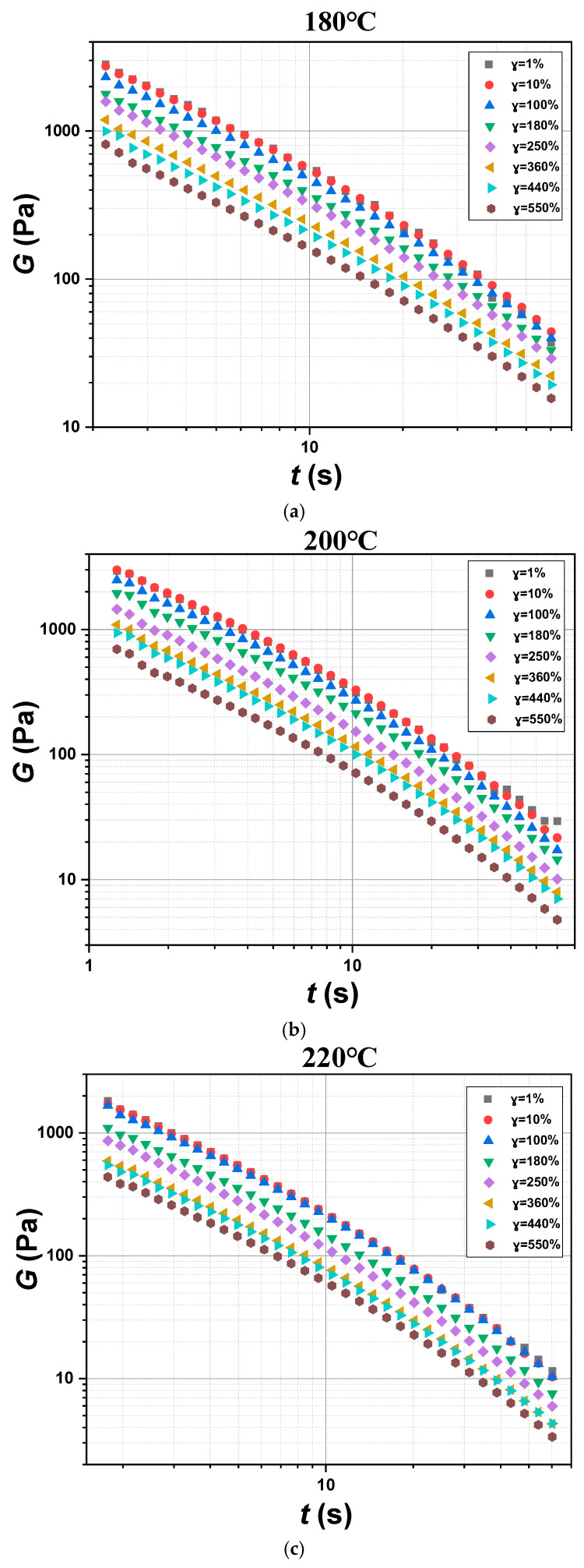
Step strain test of POE8150 at 180 °C (**a**), 200 °C (**b**), and 220 °C (**c**).

It can be observed that the relaxation modulus corresponding to strains of 1% and 10% in Figure 8 basically overlap, and the relaxation modulus only decreases with time. The relaxation modulus decreases with the increase in shear strain when the strain exceeds 30%. This is because the molecular chains of polyolefin elastomer POE8150 gradually lose their entropy elasticity during the deformation process, resulting in a decrease in the modulus with increasing strain.

### 3.2. Fitting of Constitutive Equations for POE Melt

**Figure 9:**
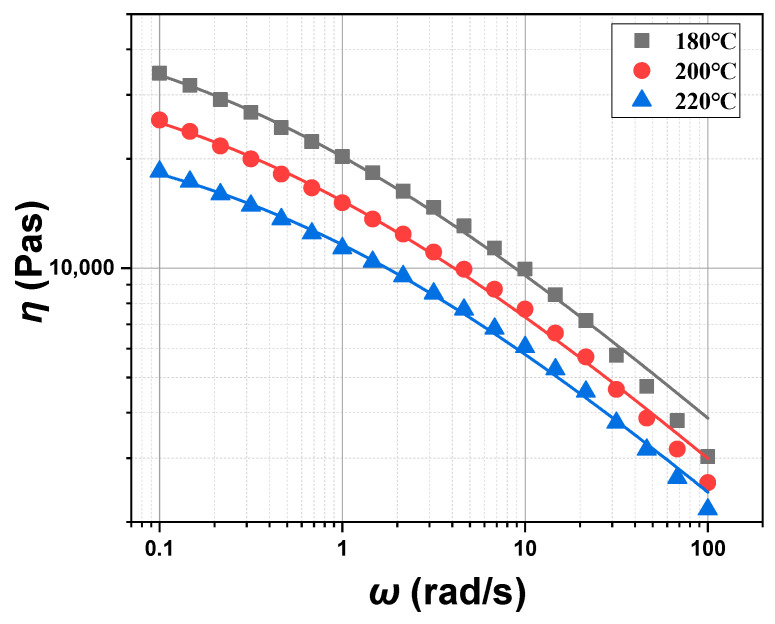
Cross model fitting rheological data at 180 °C, 200 °C, and 220 °C.

Fitting the rheological data of the POE melt using the Cross model, as shown in Figure 9.

The parameters of the Cross model were given in Table 1.

**Figure 10:**
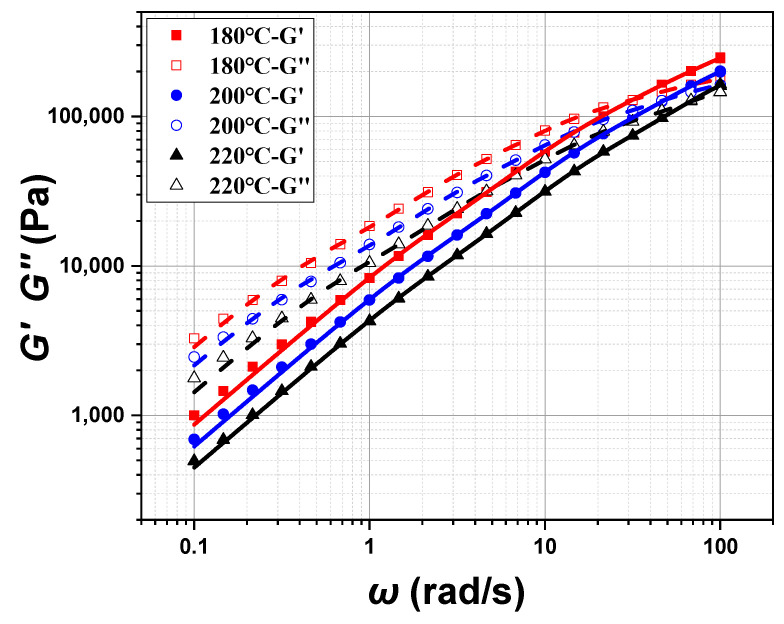
Fitting of *G′* and *G″* at 180 °C, 200 °C, and 220 °C.

The storage modulus (*G′*) and the loss modulus (*G″*) of the POE8150 have been obtained by the SAOS program. By fitting the storage modulus (*G′*) and the loss modulus (*G″*), the relaxation time *λ*_i_ and relaxation modulus *g*_i_ at different temperatures can be obtained. Figure 10 shows a good agreement between the experimental data and the estimated data. The eight sets of parameters of the relaxation spectra were given in Table 2.

**Figure 11:**
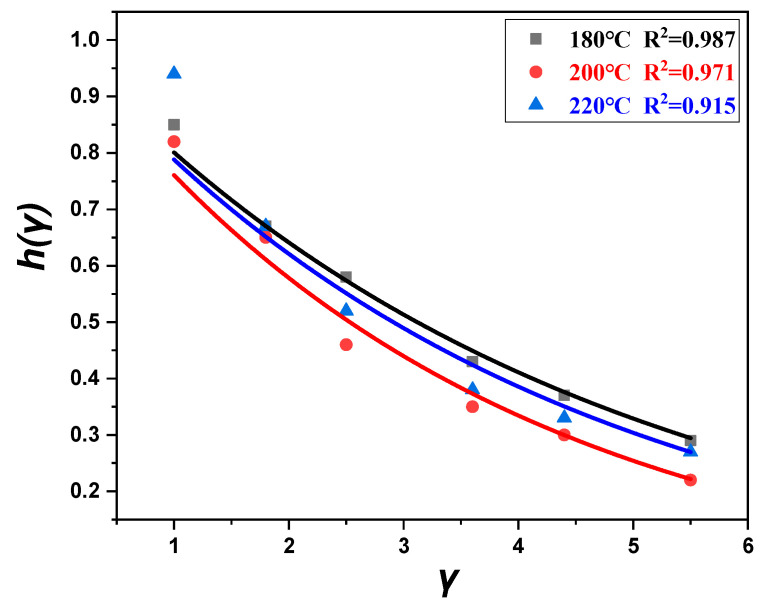
Fitting of damping function at 180 °C, 200 °C, and 220 °C.

According to Equation (6), the damping function $h\left(\gamma \right)$ was obtained by dealing the relaxation modulus under different strains obtained from the step strain tests, and the model parameter *α* of the damping function of POE8150 at different temperatures was fitted according to Equation (7), as shown in Figure 11. The parameter α of the damping function at different temperatures was given in Table 3.

When the damping function follows Wagner equation, the viscosity (*η*) of a viscoelastic fluid can be calculated as

(8) $$\eta \left(t,{\dot{\gamma }}_{0}\right)=\frac{\tau }{\dot{\gamma_{0}}}=\sum_{i=1}^{n}\frac{g_{i}\lambda_{i}}{{\left(1+\alpha {\dot{\gamma }}_{0}\lambda_{i}\right)}^{2}}\left[1-e^{-t_{r,i}}\left(1-\alpha {\dot{\gamma }}_{0}\lambda_{i}t_{r,i}\right)\right]$$

(9) $$t_{r,i}=\frac{t}{\lambda_{i}}+\alpha {\dot{\gamma }}_{0}t$$

**Figure 12:**
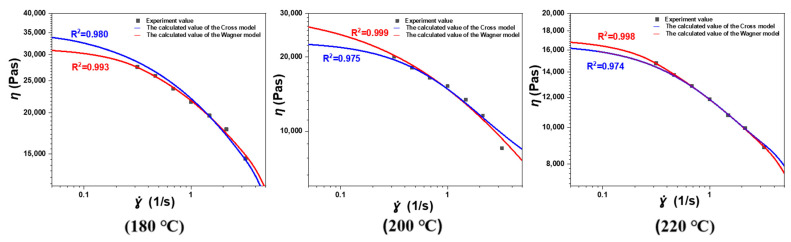
Fitting experimental rheological data at different temperatures with Wagner model and Cross model.

The fitting results of the Cross model and Wagner model on the sample experimental values are shown in Figure 12. It can be observed that both models can effectively predict the shear thinning behavior of polyolefin elastomer (POE). The Wagner model also takes into account the elastic behavior of polyolefin elastomer (POE) and can quantify the relaxation modulus damping behavior of molecular chains under high shear strain through damping functions, which is incomparable to the Cross model.

### 3.3. Differences in Pressure Drop Simulated by Two Models in the Empty Tube

**Figure 13:**
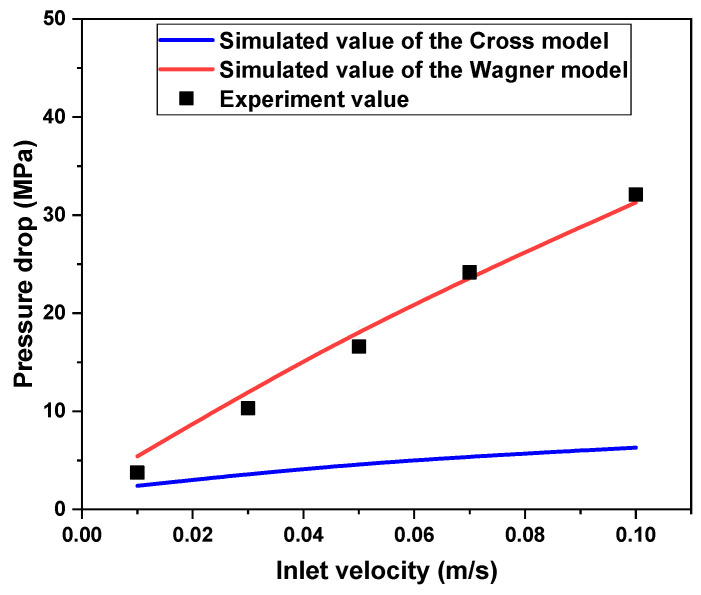
The pressure drop of experimental values and simulated values of the Wagner model and Cross model under different inlet velocities (the diameter of the tube is 33 mm).

**Figure 14:**
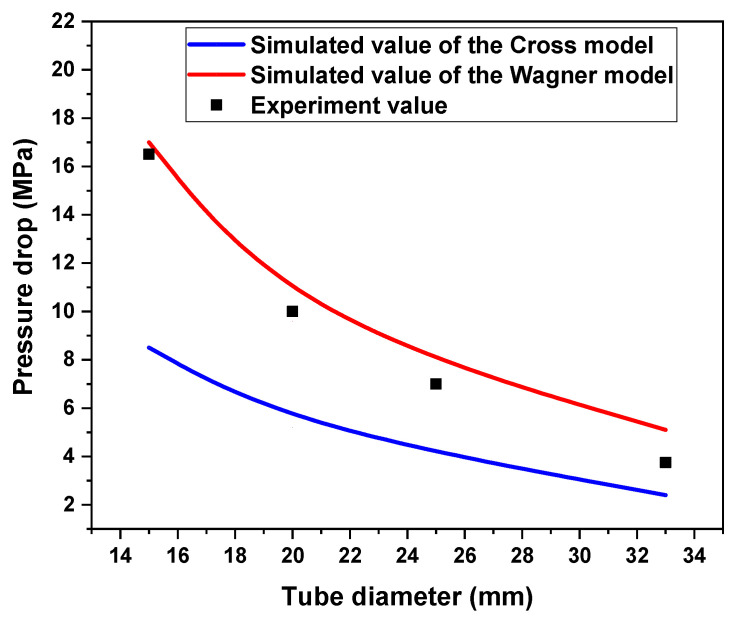
The pressure drop of experimental values and simulated values of the Wagner model and Cross model under different tube diameters (the inlet velocity is 0.01 m/s).

The pressure drop of the fluid in the empty tube simulated by the Cross model and Wagner model was compared with the experimental values, as shown in Figure 13 and Figure 14. It can be found that the Wagner model’s simulation results can better predict the pressure drop of real fluid flow in pipelines. The result shows that under the same conditionals, the pipeline pressure drop of the viscoelastic fluid is several times or even tens of times greater than that of the viscous fluid. This is because the pressure drop of the viscous fluid mainly comes from viscous resistance and viscous dissipation, and the pressure drop of the viscoelastic fluid mainly comes from viscous resistance, viscous dissipation, elastic energy storage, and elastic hysteresis dissipation. The elasticity leads to a higher pressure drop.

### 3.4. Differences in Velocity Field Simulated by Two Models in the Empty Tube

In order to explore the effect of elasticity on the laminar boundary layer in pipes, the flow of a viscous fluid and viscoelastic fluid in empty pipeline has been simulated. The length of simulated pipeline is 1 m, and the inner diameter is 22 mm. The physical quantities’ radial distribution at different lengths from the inlet was selected to study the difference of fluid flow inside the pipeline simulated by the Wagner model and Cross model.

**Figure 15:**
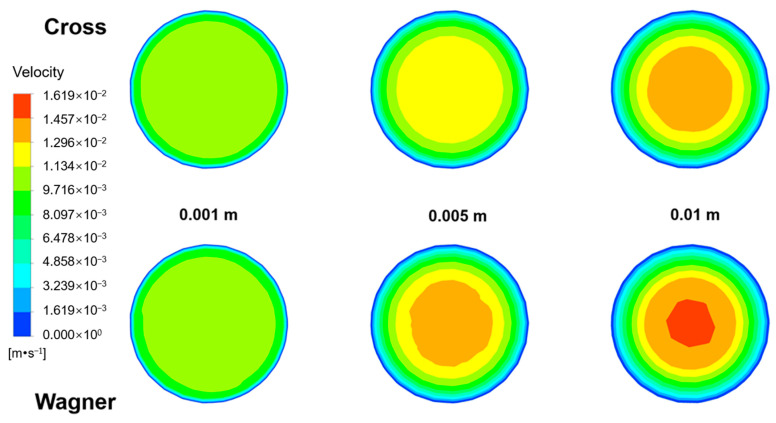
The velocity field of the fluid at different lengths from the inlet in an empty tube simulated by the Wagner model and Cross model.

**Figure 16:**
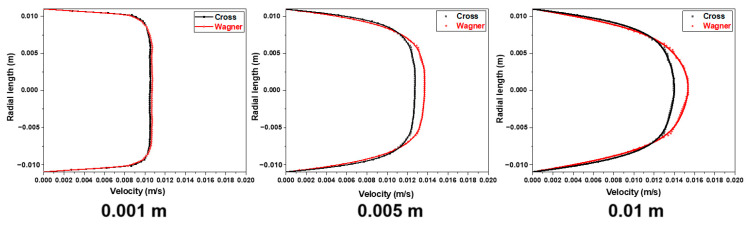
The radial velocity distribution of the fluid simulated by the Wagner model and Cross model at different lengths from the inlet.

Figure 15 shows the velocity field of fluid flow at different lengths from the inlet in an empty tube simulated by two models under the same conditions, the inlet velocity of fluid is 0.01 m/s. Figure 16 shows the radial velocity distribution of Figure 15. The velocity distribution of different lengths from the inlet reflects the development process of the laminar boundary layer of two fluids inside the pipe. It can be observed that the velocity of the viscoelastic fluid near the wall is lower than that of viscous fluid. The maximum shear rate in the pipeline flow occurs on the pipe wall, and the elastic effect will cause a certain rebound effect of the fluid near the pipe wall, which will delay the velocity of the fluid near the pipe wall. This also means that viscoelastic fluids will have a thicker laminar boundary layer. However, with the same feed flow rate, the velocity of the viscoelastic fluid at the center of the pipe is relatively higher. With the increase in flow distance, the velocity difference between the two fluids at the center of the pipe also becomes larger until reaching a steady state. The viscoelastic fluid will have a longer residence time near the wall but a shorter residence time at the center of the pipe. This will be detrimental to the stability of exported products during the production process.

**Figure 17:**
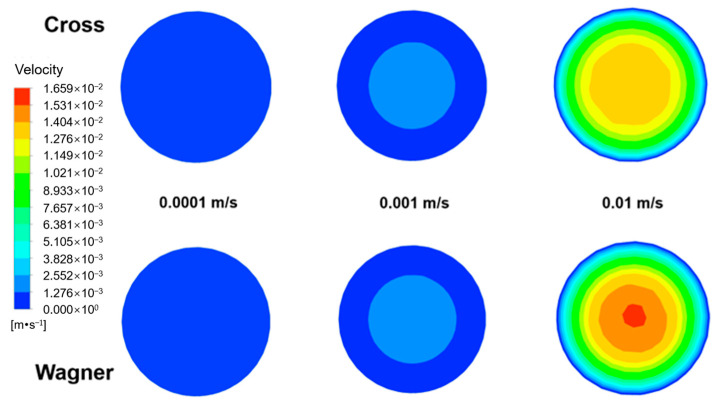
The velocity field of the fluid simulated by the Wagner model and Cross model at different inlet velocities (0.0001 m/s, 0.001 m/s, and 0.01 m/s) and the distance of 0.01 m from the inlet.

**Figure 18:**
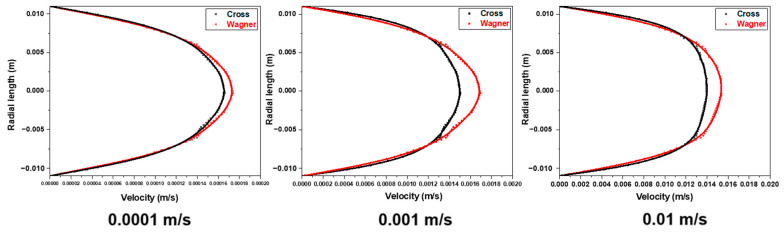
The radial velocity distribution of the fluid simulated by the Wagner model and Cross model at different inlet velocities (0.0001 m/s, 0.001 m/s, and 0.01 m/s) and the distance of 0.01 m from the inlet.

**Figure 19:**
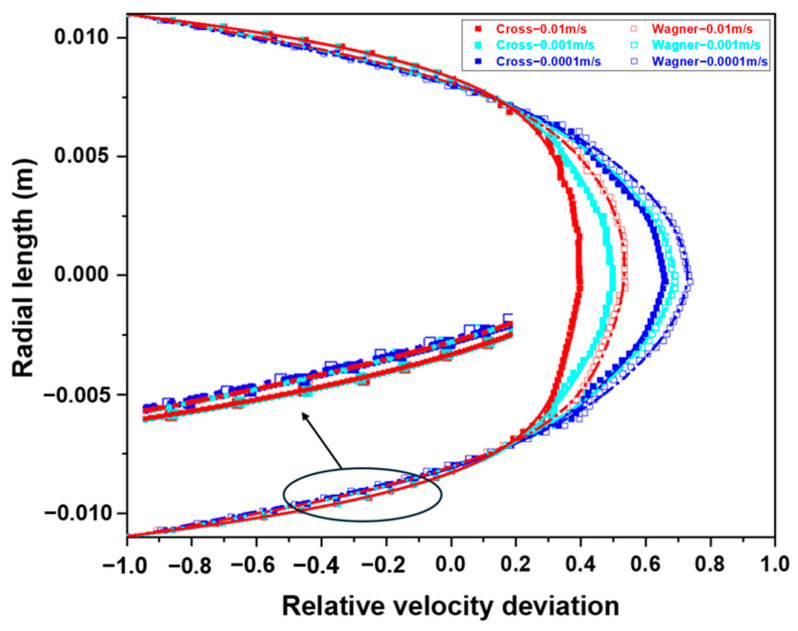
The relative velocity deviation at the distance of 0.01 m from the inlet at different inlet velocities (0.0001 m/s, 0.001 m/s, and 0.01 m/s).

Figure 17 shows the radial velocity distribution of two fluids at different inlet velocities (0.0001 m/s, 0.001 m/s, and 0.01 m/s) and the distance of 0.01 m from the inlet. Figure 18 shows the radial velocity distribution of Figure 17. It can be observed that at different inlet velocities, the velocity of the viscoelastic fluid near the wall is lower than that of the viscous fluid, and the viscoelastic fluid has a larger velocity gradient in the radial direction. Both viscoelastic and viscous fluids exhibit typical laminar flow state. We define the relative velocity deviation as follows:

relative velocity deviation = (fluid velocity − inlet velocity)/inlet velocity;

By calculating the relative velocity deviation, the thickness of the laminar boundary layer can be determined. Figure 19 shows the relative velocity deviation at the distance of 0.01 m from the inlet at different inlet velocities (0.0001 m/s, 0.001 m/s, and 0.01 m/s) in Figure 18. It can be observed that increasing the inlet velocity will decrease the thickness of the laminar boundary layer. In addition, due to the energy storage characteristic and solid-like behavior of a viscoelastic fluid, it can also be found that the variation of boundary layer thickness of the viscoelastic fluid is smaller before and after increasing the inlet velocity. When the inlet velocity is 0.0001 m/s, the boundary layer thickness of the viscoelastic fluid is about 3% greater than that of a viscous fluid, while when the inlet velocity is 0.01 m/s, the boundary layer thickness of the viscoelastic fluid is about 12% greater than that of a viscous fluid. Higher inlet velocity will lead to the more significant difference in the boundary layer thickness between the viscoelastic fluid and viscous fluid, and it will also reduce the radial velocity gradient.

### 3.5. Differences in Temperature Field Simulated by Two Models in the Empty Tube

**Figure 20:**
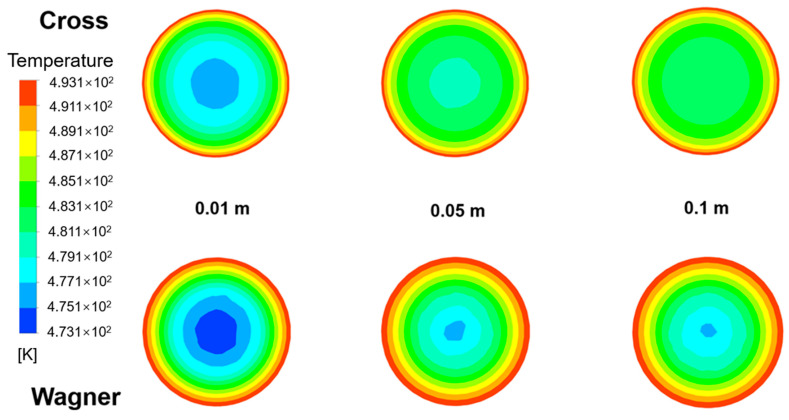
The temperature field of the fluid at different lengths from the inlet in an empty tube simulated by the Wagner model and Cross model.

**Figure 21:**
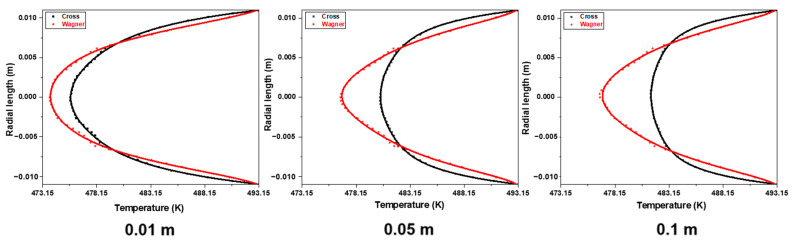
The radial temperature distribution of the fluid simulated by the Wagner model and Cross model at different lengths from the inlet.

Figure 20 shows the temperature field of fluid flow at different lengths from the inlet in an empty tube simulated by two models under the same conditions. The inlet velocity of the fluid is 0.0001 m/s, the heating temperature of the pipe wall is 220 °C, and the inlet temperature of the fluid is 200 °C. Figure 21 shows the radial temperature distribution of Figure 20. It can be seen that due to the lower velocity of the viscoelastic fluid near the pipe wall, the temperature of the viscoelastic fluid near the wall is higher under the same heating conditions, while due to the higher flow velocity of the viscoelastic fluid at the center of the tube, the temperature of the viscoelastic fluid at the center of the tube is lower. The radial temperature gradually increases with the increase in flow distance, and the temperature difference between the viscoelastic fluid and the viscous fluid also gradually increases at the center of the pipe.

**Figure 22:**
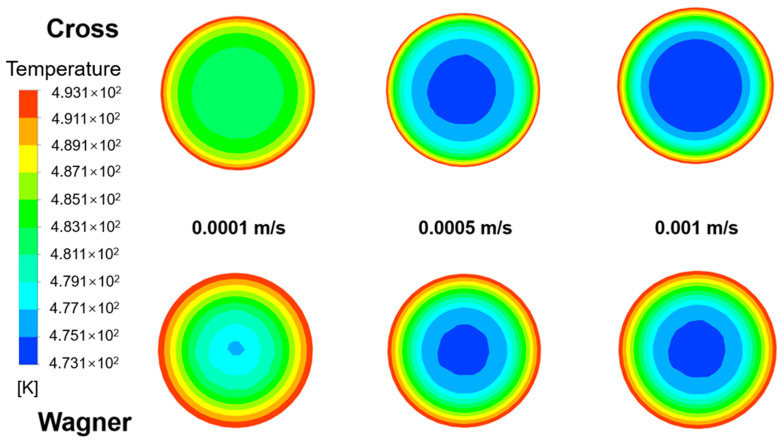
The temperature field of the fluid simulated by the Wagner model and Cross model at different inlet velocities (0.0001 m/s, 0.0005 m/s, and 0.001 m/s) and the distance of 0.1 m from the inlet.

**Figure 23:**
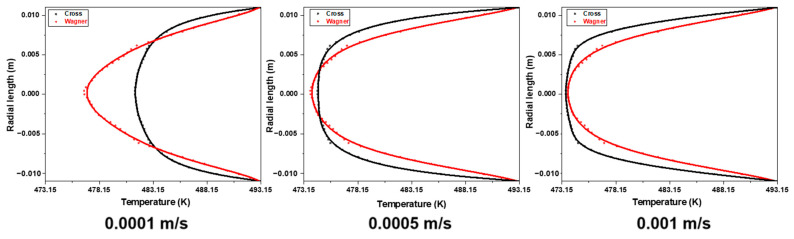
The radial temperature distribution of the fluid simulated by the Wagner model and Cross model at the distance of 0.1 m from the inlet at different inlet velocities (0.0001 m/s, 0.0005 m/s, and 0.001 m/s).

Figure 22 shows the radial temperature distribution of fluids simulated by two models at different inlet velocities at the distance of 0.1 m from the inlet. It can be found that the radial temperature at the same position decreases with the increase in inlet velocity. Figure 23 shows the radial velocity distribution of Figure 22. With the decrease in inlet velocity, the fluid temperature difference between the viscoelastic fluid and viscous fluid at the center of the tube becomes bigger, and the increasing trend of viscous fluid temperature is more obvious. When the inlet velocity decreases from 0.001 m/s to 0.0001 m/s, the temperature of the viscoelastic fluid at the center of the tube increases by 3.2 K, while the temperature of the viscous fluid at the center of the tube increases by 7.9 K. Due to the thicker laminar boundary layer of viscoelastic fluids, the radial temperature gradient of viscoelastic fluids is greater, and the temperature of the pipe wall is difficult to quickly transfer to the center of the pipe. When the inlet velocity is 0.0001 m/s, the radial temperature difference of the viscoelastic fluid is about 40% higher than that of the viscous fluid. It is also difficult to improve this situation by increasing the inlet velocity. Therefore, the heat transfer effect of the viscoelastic fluid inside the tube is worse than that of the viscous fluid.

### 3.6. Differences in Mixing Field Simulated by Two Models in the Static Mixer

**Figure 24:**
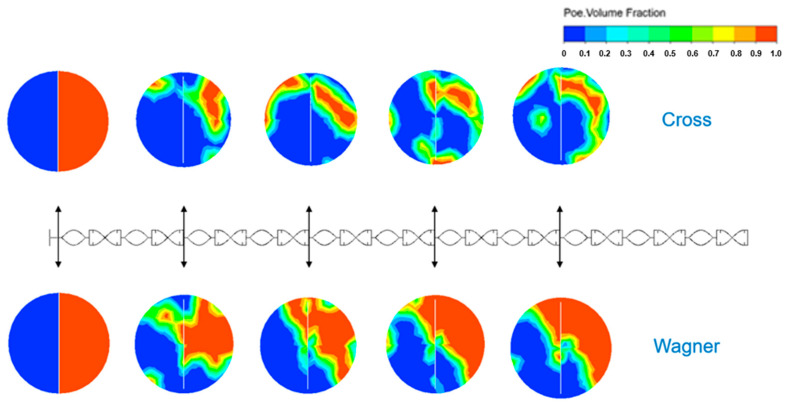
Volume fraction distribution of polymer fluid (red) and tracer (blue).

We simulated the mixing process of two fluids and a tracer in an SK static mixer. The tracer is a Newtonian fluid with the same density as the POE fluid, and its viscosity is the zero shear viscosity of the POE melt. The mixing mode of the two fluids and the tracer is heterogeneous. The inlet velocities of the polymer fluid and tracer are both 0.1 m/s, and the inlet areas of the polymer fluid and tracer are the same. The volume fraction distribution of the polymer fluid and tracer is shown in the Figure 24.

After a series of separation and recombination of SK static mixing units, the polyolefin elastomer fluid simulated by the two models formed different dispersion modes with the tracer. It can be found that the viscoelastic fluid and tracer still occupy half of the pipe, and it is similar to the initial state before mixing. The viscoelastic fluid and tracer cannot be mixed well in an SK static mixer. After separation and mixing in the SK static mixer, the fluid simulated by the Cross model and the tracer can be dispersed well. The volume fraction is very different from the state of the inlet. The mixing effect of the viscous fluid and tracer in the SK static mixer is better. This is due to the influence of elasticity, which causes viscoelastic fluids to exhibit solid-like behavior and be difficult to separate by stretching. Due to the more pronounced solid-like behavior of the viscoelastic fluid, the splitting/stretching mechanism has changed, resulting in a decrease in mixing efficiency compared to viscous fluids. It is difficult to effectively disperse viscoelastic fluids with simple SK static mixer units.

**Figure 25:**
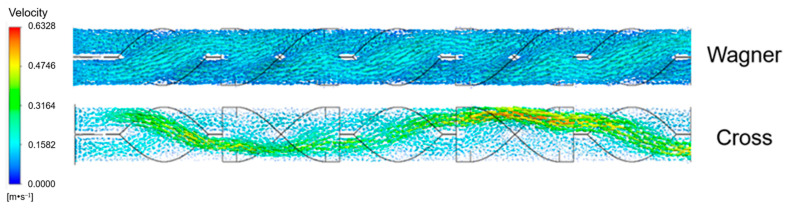
Velocity field of polymer fluid and tracer in SK static mixer.

Combining the velocity fields of the two fluids and tracer as shown in Figure 25, it can be found that the velocities of the viscoelastic fluid and tracer on both sides of the SK static mixer unit are basically the same, and the SK static mixer does not cause significant turbulence between the viscoelastic fluid and tracer, making it difficult to effectively mix. While the velocities of the viscous fluid and tracer on both sides of the SK static mixer unit are quite different. Due to the velocity difference of the viscous fluid and tracer on both sides of the SK static mixer unit, the side with the faster velocity can invade the other side more easily, which can cause turbulence in the flow field, so the viscous fluid and tracer can be mixed better.

**Figure 26:**
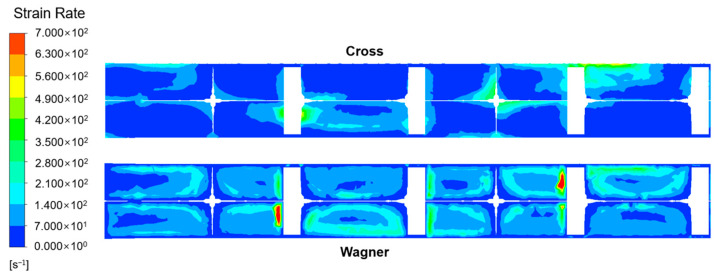
Strain-rate field of polymer fluid and tracer in SK static mixer.

By comparing the strain rate field of the viscoelastic fluid and viscous fluid as shown in Figure 26, it can be found that the viscoelastic fluid experiences a higher strain rate in the SK static mixer. Due to the solid-like behavior caused by elasticity, the viscoelastic fluid will experience stronger forces at the same inlet velocity and generate a higher strain rate while resisting the forces.

In order to improve the mixing effect of viscoelastic fluids, it is necessary to develop static mixers that can effectively peel off the boundary layer and enhance the dispersion effect of viscoelastic fluids.

## 4. Conclusions

This paper takes typical polyolefin elastomers as the research object and uses the generalized the Newtonian fluid constitutive equation, the Cross model, and the viscoelastic fluid constitutive equation, the Wagner model, to simulate the flow process of a viscous fluid and viscoelastic fluid in pipes, respectively. The differences in pressure drop, velocity field, temperature field, and mixing characteristics of fluids with different rheological properties in pipes are discussed, and it is proven that the elasticity of high viscosity polymers has an important impact on heat and mass transfer.

Although both the Wagner model and Cross model can describe the viscosity characteristics of polyolefin elastomer, the Cross model only considers the shear thinning characteristics of polyolefin elastomer (POE). The Wagner model also considers the elastic effect of polyolefin elastomers, resulting in significant differences in pressure drop between viscoelastic fluids and viscous fluids, and the pipeline pressure drop of the viscoelastic fluid is higher. Under the same conditionals, the pipeline pressure drop of the viscoelastic fluid is several times or even tens of times greater than that of the viscous fluid. The Wagner model can predict pipeline pressure drop better.

The elastic effect will cause a certain rebound effect of the fluid near the pipe wall, which will delay the velocity of the viscoelastic fluid near the pipe wall. Therefore, the velocity of the viscoelastic fluid near the pipe wall is lower than that of the viscous fluid. In addition, the laminar boundary layer of the viscoelastic fluid is thicker, and a higher inlet velocity will lead to the more significant difference in the boundary layer thickness between the viscoelastic fluid and viscous fluid. When the inlet velocity is 0.0001 m/s, the boundary layer thickness of the viscoelastic fluid is about 3% greater than that of the viscous fluid, while when the inlet velocity is 0.01 m/s, the boundary layer thickness of the viscoelastic fluid is about 12% greater than that of the viscous fluid.

Due to the slower velocity of the viscoelastic fluid near the pipe wall, the temperature of the viscoelastic fluid near the pipe wall is higher under the same heating conditions. Due to the thicker laminar boundary layer of viscoelastic fluids, the radial temperature gradient of viscoelastic fluids is greater. When the inlet velocity is 0.0001 m/s, the radial temperature difference of the viscoelastic fluid is about 40% higher than that of the viscous fluid. The heat transfer effect of the viscoelastic fluid inside the tube is worse than that of the viscous fluid.

Because of the more pronounced solid-like behavior of the viscoelastic fluid, the splitting/stretching mechanism has changed, resulting in a decrease in mixing efficiency compared to the viscous fluid.

This paper studies the effect of elasticity on heat and mass transfer of a viscoelastic fluid through a CFD simulation. Further research should include the influence of the strength of elasticity on the heat and mass transfer and the development of static mixer, which is suitable for efficient heat and mass transfer of a viscoelastic fluid.

## Figures and Tables

**Table 1:** Parameters of Cross model.

*T/*°C	*η*_0_/Pa·s	*η_∞_*/Pa·s	*λ/*s	*m*
180	77,430.7	0	18.910	0.35512
200	56,063.8	0	16.558	0.35512
220	35,867.3	0	8.489	0.35512

**Table 2:** The relaxation time and relaxation modulus for the POE8150 polymer at temperatures of 180 °C, 200 °C, and 220 °C.

i		*λ_i_*			*g_i_*	
	180 °C	200 °C	220 °C	180 °C	200 °C	220 °C
1	1.00 × 10^−5^	1.00 × 10^−5^	1.00 × 10^−5^	1.00 × 10^6^	1.00 × 10^6^	1.01 × 10^6^
2	1.00 × 10^−3^	0.0010	1.07 × 10^−3^	2.46 × 10^5^	1.00 × 10^5^	2.93 × 10^5^
3	8.65 × 10^−3^	0.0093	0.010	1.03 × 10^5^	9.98 × 10^4^	8.10 × 10^4^
4	8.67 × 10^−3^	0.0093	0.011	1.04 × 10^5^	9.98 × 10^4^	8.11 × 10^4^
5	0.028	0.023	0.069	7.61 × 10^4^	9.90 × 10^4^	4.61 × 10^4^
6	0.087	0.027	0.37	6.44 × 10^4^	9.90 × 10^4^	1.16 × 10^4^
7	3.84	0.072	2.90	4719.00	9021.00	2951.00
8	0.43	1.66	0.024	1.98 × 10^4^	9410.00	1.58 × 10^4^

**Table 3:** The fitting parameter *α* of the damping function.

*T*/°C	*α*
180	0.222
200	0.274
220	0.238

## Data Availability

The raw data supporting the conclusions of this article will be made available by the authors on request.

## Nomenclature

The following symbols are used in this manuscript:

*η*	The viscosity of the fluid
*η*_0_	The viscosity of the first Newtonian region
*η_∞_*	The viscosity of the second Newtonian region
*λ*	The characteristic time, which is directly related to the material properties
*m*	The fluid behavior index, which is related to the material properties
$\dot{\gamma }$	The shear rate
*γ*	The strain of the fluid
*m*(*t* − *t′*)	The memory function, which is time derivative of relaxation modulus
*h*(*γ*)	The damping function, which can be used for describing the nonlinear rheological response
*γ*(*t*, *t′*)	The deformation history from time t′ to t
*gi*	The relaxation modulus
*λi*	The relaxation time
*N*	The number of the Maxwell models
*G′*(*ω*)	The storage modulus of the fluid
*G″*(*ω*)	The loss modulus of the fluid
*G*(*t*)	The linear stress relaxation modulus
*G*(*t*,*γ*)	The nonlinear stress relaxation modulus
*γc*	The critical strain of the fluid
*α*	The fitting parameter of the damping function
*ω*	The oscillation frequency
